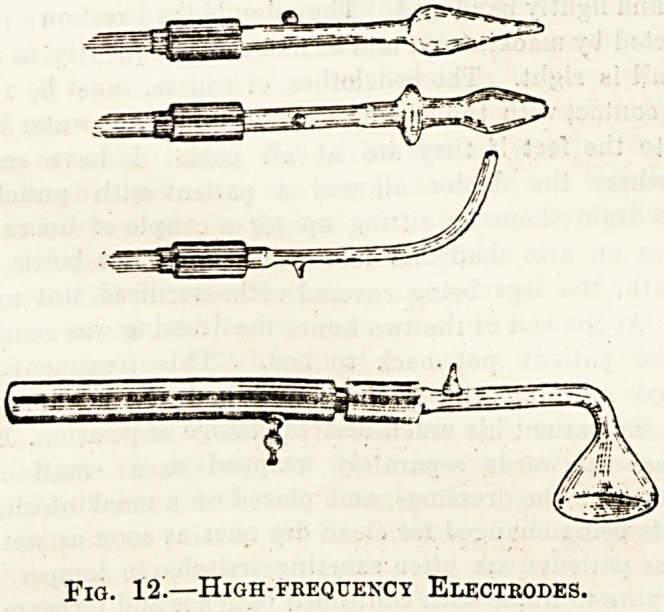# The Hospital. Nursing Section

**Published:** 1905-09-09

**Authors:** 


### The Hospital.

**Nursing Section.**

Contributions for this Section of "The Hospital" should be addressed to the Editor, "The Hospital" Nursing Section, 28 & 29 Southampton Street, Strand, London, W.C.

No. 989.—Vol. XXXVIII. SATURDAY, SEPTEMBER 9, 1905.

### Notes on News from the Nursing World.

### THE WORK OF THE NURSES AT WITHAM.

The account, by our special correspondent, of the manner in which all the nurses who happened to be available at Witham on Friday last, the day of the terrible accident to the Cromer express, came to the rescue will be read with interest. The matron of the St. Albans Diocesan Nursing Institution, Miss Flood-Jones, was away on holiday at the time, but in her absence the temporary matron, Miss R. B. Crittall, and the members of the staff, Nurses Whitehouse, Squibb, Willings, and Cannell, worked valiantly. They received valuable assistance from Nurse Luard, of the Sussex County Hospital; Nurse Easton, the cottage nurse at Witham; Nurse Barwell, of Witham; and the district nurse from Hatfield Peverel. We think that even the pessimists who say that we are a nation of degenerates, if any of them read our correspondent's account, will concede that there was not much to prove the accuracy of their theory at Witham on September 1st.

### RUSSIAN PRAISE OF JAPANESE NURSES.

The high opinion of the world in general respecting the ability and the devotion of the Japanese nurses is warmly endorsed by a Russian midshipman in a letter to his father, a well-known admiral, which has been printed in a Russian journal. He is at present in the hands of the Japanese at Kokura, a wounded prisoner. Having spoken in warm terms of the doctors, he says of the nurses: "I never imagined that they could treat me with such tenderness. After the operation they never left me, day or night. Perhaps only a mother could be so attentive. What wounds there are here! But no matter how repulsive they are, the nurses do not neglect them. They wash them themselves, and do not leave it to sanitary officers, as is the case with us. There are wounded here who had been treated under the Russian Red Cross and who say that our sisters only give medicine and drink and then go away. But all look up to the Japanese nurses with gratitude." It may be hoped that one result of the war, now happily brought to a close, will be a revision of the Russian Red Cross system, which, with all the admirable services rendered by individuals, is clearly in urgent need of reform.

### LIFE IN NURSING HOMES.

A patient, commenting upon her rapid recovery and short stay in a nursing home, was much amused by the rejoinder of the nurse: "We see plenty of life here. It either goes one way or the other, and that very quickly."

### AN ISOLATION HOSPITAL WITHOUT A TRAINED NURSE.

A correspondent makes a startling statement in a letter which we publish to-day. She says that there is an isolation hospital in Northamptonshire which contains 80 beds, and is not only without a trained matron, but does not possess a trained nurse. In fact, she names the institution, to which she was herself attached for nearly two years. The urgent importance of a trained nurse in an isolation hospital containing 80 beds does not need to be argued, but our correspondent is able to prove from her own experience the danger of only untrained persons being in attendance on the patients. On several occasions, she affirms, she had to get up in the night to different patients, and in one instance to give constant attendance to a tracheotomy case, the assistant nurse not knowing how to clean the tube. The isolation hospital to which she alludes is not under the jurisdiction of Poor-law Guardians; but as the absence of trained nurses involves risk both to the patients and, in a less degree, to the community, action should at once be taken by residents in the locality who can bring influence to bear with the view of altering a condition of affairs which constitutes a grave reflection on the county administration.

### FALSE ECONOMY AT STOKE.

The Stoke-upon-Trent Guardians have decided to advertise for probationer nurses beyond the boundary of the union. This is no doubt a necessary step, as employment in the workhouse infirmary is often unattractive to those dwelling in its locality. But this step alone will not, in our opinion, secure the desired number of candidates. Nowadays the would-be nurse requires that the training she is about to enter into shall be such as shall render her an eligible candidate for good nursing posts elsewhere. Moreover, workhouse infirmaries do not appeal to well-to-do nurses, and as a rule they would rather pay for the privilege of the best training and experience at one of the large training schools. It is therefore to the less well-to-do nurses that the ordinary workhouse infirmaries must look in order to recruit their staff. But to such, a salary, however small, is necessary. The Stoke Guardians, however, offer no salary until the second year of service. They have also reduced the number of staff nurses, so that more work with less supervision will be the lot of the probationers—a condition which cannot improve the standard of training. We believe that excellent experience and training can be obtained in a large workhouse infirmary, but this can only be the case when the Guardians acknowledge the claims of their nurses in return for the services they render. For the sake of efficiency we hope that the Stoke Guardians will be induced to offer better conditions of service, and we feel sure that the popularity which would result would remove all difficulty in obtaining a sufficiency of suitable candidates.

### THE QUESTION OF SHORTER SKIRTS.

At the ninth conference held on Saturday at Lytham of the members of the North-Western District of the St. John Ambulance Brigade there was an interesting discussion as to a proposed reform "in the dress of the sisters of the nursing divisions." It was agreed that for the future the bonnet strings should be of white lawn ribbon, four and a half inches wide; that the apron—which at its edge must be four inches from the ground—should be made of plain linen with two pockets on the outside, right and left, and always worn by the nurse when engaged in public duty. It was also decided that the edge of the dress skirt should be two inches from the ground. This last regulation we should like to see made one of the rules of every hospital and every nursing association, district or private, throughout the kingdom. The enforcement of such a rule would reduce the risk of nurses carrying infection to others to a minimum; the chances of nurses catching cold owing to wet skirts on a damp day would be materially lessened; and the reproach which is now frequently levelled at nurses that they appear in untidy skirts frayed at the bottom by constant friction on unsanitary pavements would be removed. It is strange that a woman who understands her business should need to be told that a skirt which well clears the ground is essential for a nurse when she is on duty.

### A MATTER OF FORETHOUGHT.

An assistant nurse at Newton Abbot Workhouse Infirmary, who was leaving for the purpose of obtaining further certificates, applied to be allowed to leave a week earlier than the expiry of her notice. As she was entitled, according to the scale, to three weeks' holiday which she had not had this year, the house committee recommended that it be granted. Exception was taken at the usual meeting of the Guardians to the proposal, and evidently the committee intended to pay the nurse an extra week's salary in lieu of holiday. The clerk said that this could not be done and consequently the Guardians did not vote on the matter. Eventually the Guardians agreed to allow her to leave at the time she wished. It seems to us a pity that nurses should show want of business forethought which operates to their own detriment. If the nurse at Newton Abbot Infirmary had only given the matter a few minutes' consideration she would have recognised the folly of going to a new sphere of work without having enjoyed a holiday for more than a year. There would have been no difficulty in securing the usual leave from the Guardians before giving notice, whereas as it now stands, unless she has allowed for an interval between relinquishing her old duties and taking up fresh ones, she will run a serious risk of breaking down. Even if she has arranged an interval she will quite needlessly have to suffer the loss of her salary.

### REBUKING ROTHERHAM NURSES.

The Rotherham Guardians having rejected the report of their own committee of investigation, which was appointed in order to investigate complaints that have recently been made against the charge nurses in the workhouse infirmary, adopted a suggestion that the whole of the nurses, including the superintendent, should be called into the board-room, and given "a good talking to" by the chairman. The author of the suggestion affirmed that there had been tittle tattle, whispering, backbiting, and unpleasantness which ought to be suppressed, and he insisted that the staff must be made to understand that they must loyally obey the superintendent nurse. The whole of the nurses were, accordingly, sent for into the board-room, and the chairman rebuked them on the lines proposed. We hope that the rebuke will have the desired effect, and that in future the probationers will not be hindered in their studies by noise on the part of any one, and that the authority of the superintendent nurse will not be disregarded.

### NURSES AS INSPECTORS OF MIDWIVES.

It is interesting to observe that the local authorities continue to choose fully-trained nurses for the post of inspectors of midwives. Miss Swain, who has just been appointed inspector for East and West Suffolk, answers this description, and she has had, in fact, several years of experience in all branches of nursing. We shall be glad to announce any appointments that may be made to inspectorships, whether of trained nurses or qualified midwives.

### THE HOLT-OCKLEY SYSTEM AND DISTRICT NURSING.

We do not quite understand the tactics of the promoters of the Cottage Benefit Nursing Association. There is already at work in Tetbury a District Nursing Association, whose work is admittedly excellent. But there has just been held at Tetbury a meeting to advocate the extension of the Holt-Ockley system to that town and district. We agree that the society, founded by Miss Broadwood, has been of good service in the past, and may still be useful. But we do not agree with its intrusion into localities where trained nurses are already doing their best to minister to the sick. Apparently, the idea of those who support this policy is that, there not being room for two organisations, it would be wise to oust the trained district nurse and set up in her place the half-trained cottage nurse. In our opinion a movement of this kind is calculated to do harm rather than good.

### AN INSTITUTION WHICH DOES NOT ISSUE A REPORT.

We learn that the Bedfordshire Hospital Trained Nurses' Institute at Bedford has ceased to issue a report. This is a very strange attitude for an organisation with a committee of management to take up. The president is Mr. S. Whitbread, and the committee consists of twenty-five members. The institute was started in 1889 and depended to a considerable extent, at any rate for a time, upon the contributions of the public. We are glad that it has become self-supporting, but, as it is not a private organisation, the authorities are not absolved from the obligation of issuing a report.

### QUEEN ALEXANDRA'S MILITARY NURSING SERVICE.

We are officially informed that the following staff nurses in Queen Alexandra's Imperial Military Nursing Service have been appointed sisters:—Miss F. G. P. de S. Zrinyi, Miss H. M. Drage, Miss L. M. Moor, Miss A. M. Pagan, Miss M. E. Richardson, Miss L. M. Toller, Miss L. F. A. Waller, and Miss A. Willes. The appointments of Miss L. Cunninghame, Miss V. C. Paschali, and Miss D. J. Saunder, as staff nurses have been confirmed. Miss M. Barton, staff nurse, has been transferred from Cork to the Military Hospital, Chatham; Miss N. C. Johnston from Devonport to the Cambridge Hospital, Aldershot; Miss M. M. McCreery has been posted to the Royal Victoria Hospital, Netley; and Miss L. M. Draper has been sent to the Royal Herbert Hospital, Woolwich.

### A DEVONSHIRE NURSE AT LYDENBURG.

Miss Fulford, daughter of the Vicar of Hennock, Devon, who has been nursing in Pretoria, Johannesburg, and other places in South Africa in the last three years, has been appointed assistant nurse by the Lydenburg Board of Control, and left Pretoria, where she was on the staff of the hospital, a few weeks since to take over her new duties.

### NURSE AND DEACONESS.

There has just passed away in the county of Durham a trained nurse who earned great distinction in another sphere of work. We refer to Miss Sarah Elizabeth Clarkson, the first Head Deaconess of the Society of Christ and the Blessed Virgin Mary, in Durham. Miss Clarkson was the daughter of an eminent Cambridge man who had been a Fourth Wrangler and Fellow and Tutor of Trinity College, was early in life influenced at Walsall by Sister Dora to become a nurse, and was subsequently trained first at the Royal Hants County Hospital, Winchester, and then at Middlesex Hospital. After her training she took charge of Penbury Cottage Hospital for five years, and here she began to work with the Sisters of the Holy Name in Wednesbury. In 1887 she determined to give up nursing entirely, and joined the movement which was then being developed in Durham by Bishop Lightfoot, eventually being appointed by Bishop Westcott as first chief of the institution which is now so well known in the north. The success which Miss Clarkson achieved as a nurse was repeated in her new sphere of labour, and was mainly due to her mental gifts, her common sense, her vigour, her tenderness, and her devotion.

### GARDEN PARTY AT ALNWICK CASTLE.

On Saturday last a large garden party took place at Alnwick Castle. The Duchess of Northumberland had invited as her guests the nurses of the Northumberland County Nursing Association, numbering upwards of 60, and along with them two members of committee from each affiliated association, the party altogether being about 150. The weather was all that could be desired. Carriages met the various trains and conveyed the guests to the Castle, and by 11 o'clock all had assembled. The Duchess had a word of welcome for each one present. Miss White, the well-known county superintendent, took an active part and did much to make the day a success. Luncheon was served at 12 o'clock in the guest hall, and during the afternoon a cinematograph entertainment was given and much appreciated. The remainder of the time was spent in strolling round the beautiful grounds. Tea was at four o'clock, and shortly afterwards the company dispersed, carriages conveying them to the station. A most enjoyable day was spent, and it proved a happy reunion for many nurses who had not met since the same occasion last year.

### A MONTHLY JOURNAL IN AUSTRALIA.

The *Australasian Nurses' Journal*, which has hitherto been issued quarterly, is now published monthly. This step has been decided upon chiefly owing to the adoption of a scheme for a central examination, and the publicity for notices in connection with these examinations and information concerning them has chiefly led to the change. The *Australasian Nurses' Journal* has always maintained a high standard, and we congratulate the nurses of Australia upon a new departure which cannot fail to further the interests of all that is best in nursing throughout the Commonwealth.

### THE CONTRIBUTIONS OF PATIENTS IN HOSPITALS.

It is interesting to observe how the increased efficiency of the nursing in a hospital may affect the contributions of the patients. Since Miss Lillie was appointed matron of the North Devon Infirmary at Barnstaple the nursing staff has been increased in number by three, and the diet and comfort of both the staff and the patients has much improved in spite of the cost of provisions being £267 less for the year ending June 30th, 1905, than for the previous twelve months. For the year ending June 30th, 1904, the contributions from patients amounted to £19 2s. 8d., and for the year ending June 30th, 1905, they rose to £43 11s. 10d. The gifts in kind have become a regular feature, and patients and their friends send from their gardens fruit, vegetables, and potatoes. These figures confirm the assertion that the patients appreciate the care and attention which they receive.

### A RESERVE FUND AT ST. NEOTS.

A feature in the report of the St. Neots and District Nursing Association which was adopted at the annual meeting, was the intimation that the amount realised by the Arts and Crafts Exhibition was £56 10s. Of this sum £50 has been placed on deposit as a reserve fund. The work of the Queen's nurse during the year was exceptionally heavy, no fewer than 70 cases having been attended and 2,843 visits paid in St. Neots, Eynesbury, and Little Paxton. An attempt to reduce the size of the general committee and to make them the working committee was not successful. There does not appear to be any sufficient reason for the proposed change.

### SHORT ITEMS.

The late Mr. Hugh McAllister, of Amberley Street, Sunderland, has left £100 to the Queen Victoria Memorial Fund for the Sunderland District Nurses.

### The Nursing Outlook.

"From magnanimity, all fear above;
From nobler recompense, above applause,
Which owes to man's short outlook all its charm."

### MANNERS AND WORK.

The manner and air which accompanies an action are more expressive than words. This point is very often forgotten or fails to be appreciated by the majority of people. Yet the personal bearing of any one in authority, and especially of a nurse on duty, is often characteristic of the individual, and is taken as an index to her disposition by the wise and experienced. It is therefore very important for those in authority, and for humbler workers too, to study deportment and conduct in all the circumstances of their work. The habitual practice of courtesy, under the most trying and irritating circumstances, gives strength and authority to the worker and wins consideration, whilst it imposes restraint upon the most aggravating and inconsiderate of patients. Many technical and other matters are taught in training schools in the present day, but those probationers are most fortunate who have over them a matron, whose deportment and courtesy are proverbial, for these characteristics justly secure general confidence and recognition.

Our thoughts were carried into this channel by a visit of inspection we paid to an operation theatre on the day this article was written. Our business was to see that the operation theatre was in perfect order, and to ascertain how long it would be before it was ready to receive the surgeons and the patient. The operation sister was a portly, good-natured, sturdy woman, who no doubt knew her work and did it efficiently according to her lights. She had been worried, however, by a long operation which had occupied some three hours, and was disturbed by the fact that a second serious operation had to be commenced almost immediately. We asked her what number of operations she usually had in the theatre each day. Her reply was, "One large operation is all we expect." We tried to cheer her with the remark that that day the theatre was being made more than ordinarily useful, but she was evidently put out by the extra trouble and took no pains to conceal the fact. In a word her manner was disagreeable and uninviting. Of course a busy hospital sister may laugh at th[illegible] experience, but the very pettiness of the incident, which ruffled our friend, emphasises the importance of the habitual practice of self-control, and the cultivation of a personal bearing which is pleasant and winning.

Of course sisters and nurses, and even matrons, are often misunderstood. This point was usefully and touchingly illustrated in the incident in a nurse's life, entitled "Hearts of Stone," which we published on the 26th ult. No doubt, too, doctors and patients are at times inconsiderate, and it may be occasionally unjust, whilst the vagaries of patients friends would occasionally ruffle the patience of a saint. Yet there is no merit and no character in having a pleasant and attractive manner when the sun shines and all runs smoothly as a marriage bell. The true test is to be able to maintain the same polite deportment when the skies are dark and everything seems awry. To recognise the force of all this is to realise the true meaning of character, and to practise it conscientiously for the reason that, anything else than such conduct must be opposed to the principle of life, which every worker must recognise to be the true aim of the best and wisest of woman workers, who devote their lives to the care of the sick. It is well, too, to cultivate strenuously pleasantness of manner, because it tends to ensure smooth working with others, and thus materially aids the worker in the daily round of duty. Where those in authority are recognised as habitually exercising great patience and consideration for others, there everyone who comes in contact with them unconsciously feels the necessity of being on their best behaviour too. The love and affection which come to the worker who is invariably considerate and thoughtful for others bring with them rewards, which the best of us have found, in the course of our lives, to be priceless to the recipients.

Then again a pleasant manner, and the reputation which it carries with it, are valuable assets to a nurse in a business sense. Her patients always recognise gratefully such characteristics in a nurse, and they are certain to make her popular and to win for her the confidence of both doctors and patients throughout the whole course of her nursing career. We know, of course, how many and great are the difficulties of continually cultivating the cheerful countenance. To succeed it must have its origin in a grateful heart, grateful that is for the high privilege of rendering service to others in the happiest manner, despite all difficulties, and strong temptations to exhibit temper or even just resentment. After all, for the individual worker, when the day's duties are over and the quiet hour at last comes, it is pleasant indeed to know, that, despite all the worries and harrass of the day which is done, the nurse has succeeded throughout in exercising patience. In such circumstances she can realise with truth, that after all her self-control has been so deliberately exercised, as to have carried her through a troublesome day, without the slightest indication of the many trials she has suffered, and without allowing one of them to reveal itself, by any change of manner or act of impatience. Emerson says: "Good manners are made up of petty sacrifices." This is so greatly true, that it is well to charge the memory with it, and so to sweeten the character and make it altogether lovable.

### Medical Electricity and Light Treatment.

By KATE NEALE, Sister-in-Charge of the Actino-Therapeutic Department, Guy's Hospital.

### VII.—HIGH FREQUENCY.

IN no branch of electro-therapeutics have more rapid strides been made in recent years than in the use of high-frequency currents. It is only a few years ago that they were first found to exercise a beneficial effect, and yet to-day every complete electrical department contains the elaborate apparatus required for their generation. Whether high-frequency treatment will retain to the full the favour at present extended to it is doubted by some, but most doctors admit that the currents influence the course of certain diseases to a gratifying extent.

FIG. 10.—HIGH-FREQUENCY CUBICLE.

A glance at fig. 10, which represents the high-frequency cubicle at Guy's Hospital, will show you the complexity of the instruments required, but it is unnecessary to burden your mind with details of their mechanism. It will be enough to know why the term "high frequency" is given to the currents, and how the treatment is applied to the patient.

The current, which is usually obtained from the main, is led to an induction coil and from there conducted to two jars, known as "Leyden jars" (fig. 11, B) underneath the table. From the outer coating of these jars the current passes to the tall cylinder (C)—the **Resonator**—at the top of which is a terminal (D) while on the table are two others (EE). The object of using the jars is to obtain a current that is made and broken with enormous rapidity. With a faradic apparatus you will remember the current is made and broken some two hundred times a second, but with the apparatus just described the frequency of the currents is inconceivably more rapid, and probably amounts to something like *two million* a second. Hence the origin of the term "high frequency." There is a very interesting fact connected with the currents of high frequency. A current that oscillates with this periodicity can be passed safely through the human body, when one of the same strength but continuous would cause instant death. The harmlessness of the former current has been proved by an experiment which has a startling effect when seen for the first time. A number of people arrange themselves in two parallel rows, and each individual grasps the hands of those on either side. The end one of each row is joined to a terminal of the high-frequency apparatus, while the two at the far ends hold the wires of an electric lamp. If now the apparatus be set in action the lamp will glow, yet no current can be felt by the people through whom it is passing.

### How to Treat.

Although a high-frequency current passing through a patient produces no obvious sensation, it appears to influence the system in a marked way, as shown by the fact that during treatment the amount of carbon dioxide breathed out from the lungs is appreciably increased, and after treatment the temperature is often elevated. The usual methods of application can be summarised as follows:—I. Direct application; II. Condensation; III. Local application; IV. Enclosure of patient in a solenoid.

I. **Direct Application.**—This method is less often used than the succeeding ones. Connect two wires to the terminals EE, and to the free end of each attach an ordinary electrode, such as is used in faradism. These electrodes, which are used moistened, must be well pressed against the bare skin, and unless otherwise ordered are to be kept stationary on the parts of the body indicated by the doctor.

II. **Condensation.**—This variety differs from the preceding in that only one terminal is directly connected to the patient. On a couch place a large flat metal electrode, measuring about 36 by 12 inches,

FIG. 11.—HIGH-FREQUENCY APPARATUS.

and to it connect one of the terminals E. Cover this electrode with a thin mattress, on which the patient reclines fully dressed. Put the other terminal E in communication by a wire with a metal rod grasped by the patient. The current passing to the metal plate charges the patient through the mattress by what is known as "condensation."

FIG. 12.—HIGH-FREQUENCY ELECTRODES.

No tingling or sensation of any sort is experienced, but if the bare skin is touched a slight spark may be drawn. The treatment is usually continued for ten minutes, and requires no special precautions.

III. **Local Application.**—This cannot be given except by using the resonator. The current is led off from the terminal D at the upper end of the resonator by a wire ending in a special form of electrode. Some of the commoner forms of high-frequency electrodes are shown in fig. 12. Each consists of a hollow glass tube of varying shape ending in a metal terminal enclosed in a vulcanite insulating handle. The glass tube has usually been exhausted of air, though sometimes it is filled with water, or salt and water. On connecting the wire from the resonator terminal D to the screw of the electrode, the current (if the tube be a vacuum) passes along the empty tube, producing a violet luminescence, and if the electrode be brought near the clothing a shower of violet sparks leap out. Not infrequently a metal electrode is used, similar to the bath electrode described under Static Electricity, but with a much smaller knob. This variety is apt to produce more stinging sparks.

In treating locally you need not take any precautions to insulate the patient, and the electrode is applied in a labile fashion, either directly to the skin, or through the clothes, according to your instructions. Remember, however, that if the electrode is near but not actually touching the skin, painful sparks will pass, and this must necessarily occur when the treatment is given through the clothes.

The wire attached to the summit of the resonator is usually insulated in rubber tubing, but this is not always sufficient to prevent a slight shock being felt by any one manipulating the wire. You will therefore find it advisable to use a handle of some non-conductor such as vulcanite, about twelve inches long, provided with a hook at the end to hold the tubing.

The strength of the high-frequency current may be graduated in various ways, but the simplest is by means of the resonator. The resonator (fig. 11, c) consists essentially of a spiral coil of thick wire, the upper three-quarters of which are covered by a vulcanite top. Lying in contact with one of the lower spiral coils is a metal index shown in the figure, and by means of the handle seen on the right-hand side of the table the whole resonator can be slowly revolved so that the index travels up and down the spiral. Thus more or fewer turns of the spiral are put in the circuit and the strength of the treatment increased or decreased respectively.

Some medical men are in the habit of prescribing local and condensation treatment combined, in which case you must first join the patient to the terminals E E as described, and then treat with an electrode attached to the resonator. It sometimes happens that the current will produce a minute crack in the glass electrode, allowing air to gain access to the tube and the luminescence ceases. Such a tube is said to be "sparked" and should be returned to the makers for re-exhaustion.

IV.—**Enclosure of Patient in a Solenoid.**—Treatment by this method is expensive and cumbrous, and as it does not seem to possess any special advantage is rarely used. The solenoid is a large cylindrical wire cage inside which the patient sits. The cage is then connected to the high frequency

MEDICAL ELECTRICITY AND LIGHT TREATMENT—*Continued.*

apparatus and the patient becomes charged by condensation. Another form of solenoid is made to enclose couch and patient together, but here again the method of treatment is by condensation.

### Dangers.

The chief danger arises in local treatment when volleys of sparks are apt to pass from the metal electrode to the patient. A sharp stinging pain is produced, and the skin shows a redness which lasts some little time. The effect cannot be avoided when treating through the clothes, though it may be lessened by reducing the thickness of the garments to a minimum, for with high frequency, as with static treatment, the thicker the clothing the severer are the sparks. In applying local treatment to the skin, you can avoid sparking by holding the end of the electrode in good contact with the skin.

### Diseases Treated.

Treatment by high frequency has been known so short a time that it is difficult to discuss opinions as regards the diseases it benefits most. At present medical men are closely observing its effects over a wide range of conditions. Persistent headache, neuralgia, neurasthenia and allied conditions often respond to the treatment but even more definite and more serious morbid states such as diabetes mellitus, are believed by some to be at any rate improved by a course of treatment. More recently the currents have been used in various affections of the skin.

### The Nurses' Clinic.

### THE MANAGEMENT OF DROPSICAL CASES.—BY A MATRON.

During the year or two that I was staff nurse of a men's medical ward, I used to experience a certain heart sinking when told that a dropsical case was coming in.

Whether the primary cause is disease of the heart, kidney, or liver, the symptoms call equally for the most careful and efficient nursing, which nursing is beset, however, with many difficulties.

For instance, these patients, who really need complete rest in bed, are most unwilling to stay there, and though it is so important to avoid chill, yet their continual restlessness makes it hard to keep them warmly covered. Owing to their great weight, it is difficult to lift and settle them comfortably in bed, and, in addition, the mere fact of their being dropsical renders them peculiarly liable to bed-sores. Necessary as it is that the bowels should be kept freely open, their frequent action again leads to restlessness, and increases the likelihood of bed-sore. The amount of fluid they are allowed to drink is often restricted, a restriction which they are inclined to resent, being usually very thirsty, and when paracentesis is resorted to, or Southey's tubes are inserted in the legs, there is always the anxiety that the restless patient may pull out his tubes, or so disarrange his dressings, that the punctures become septic, and erysipelas supervene.

Hot air and vapour baths, hot packs and hot bottles, all need the greatest care in application to these patients, lest their tender skin be blistered or injured in any way.

It is well, as soon as a case of this kind is admitted, to place him at once either on a full-length water-bed, or to give him a circular air or water-cushion, so as to avoid pressure from the first on those parts likely to develop bed-sores. As dyspnœa is a symptom commonly present, a bed-rest and comfortable pillows will be needed to prop him up. All urine must be saved and measured and a specimen put up for testing as often as the physician orders. The scrotum, if very œdematous, must be supported on a small cushion made of wool and tow covered with jaconet and frequently cleansed, and it is important that these patients should be kept scrupulously clean, both in person and bed linen, the draw sheets receiving more than a usual share of attention, so as to avoid any creases or dampness.

At night, and this is especially the case when dropsy is associated with cardiac disease, these patients are apt to be most troublesome and restless. They often cannot lie in bed, and usually are allowed to sit up for a time, warmly wrapped in blankets in an arm-chair if they so desire it. The dyspnœa, being worse at night, hinders them from sleeping, and causes them to start up with a feeling of suffocation when they do drop off for a few minutes.

A night nurse with two or three such cases in her ward, finds her post no sinecure. No sooner is one patient comfortably settled, than another begins to be fidgety. The cough present in most cases is noisy and persistent, that alone being sufficient to prevent sleep.

Night always accentuates the symptoms, and if in addition, the weather be at all hot, they are many times worse.

In allowing the patient to get in and out of bed as he pleases, much care must be taken to avoid chill. His dressing-gown and slippers must be warm, and if there is no fire in the ward, he should sit by his bedside, warmly wrapped up in a blanket, his feet resting on a hot water-bottle as a footstool. Should he be allowed to go out to the lavatory, the windows must be closed while he is there. When by reason of increasing illness, or because Southey's tubes have been inserted, he is unable to get up, it is often very difficult to procure him any ease at all, and the nurse's resources are taxed to the utmost. His position needs changing often, his pillows frequent adjusting, the bed constant attention lest it become wet with the fluid oozing from the legs. These are sometimes simply punctured and left to drain, in which case, in order to keep them aseptic, they are best wrapped in sterilised gauze, covered with wood wool and lightly bandaged. They should then rest on a pillow protected by mackintosh, and be looked at frequently, to make sure all is right. The bedclothes, of course, must be raised from contact with the legs by a cradle, and a hot-water bottle kept to the feet if they are at all cold. I have seen a case where the doctor allowed a patient with punctured legs to drain them by sitting up for a couple of hours at a time in an arm-chair, his feet resting on a hot bottle in a foot-bath, the legs being covered with sterilised lint meanwhile. At the end of the two hours the dressing was renewed, and the patient put back to bed. This treatment was repeated morning and evening, and had the advantage of giving the patient his much-desired change of position. Each leg was afterwards separately wrapped in a small clean blanket, over the dressings, and placed on a mackintosh, the blankets being changed for clean dry ones as soon as wet.

These patients are often exacting, irritable in temper, and very trying to nurse with continued patience and forbearance. Also their appetite is capricious, their digestion bad, and they not infrequently have attacks of vomiting. They should never be left alone, or excited, or allowed to make the least unnecessary exertion. Symptoms of uræmia should be watched for, and the action of any drug that they may be taking carefully noted.

### The Woman Worker.

### JUSTICE FOR HERSELF AND A WORD TO HER FAMILY.

Some women have worked in all ages, but few women in this country until recent years have worked successfully for a living. Success in this connection is of course a word of relative meaning, which for the present purpose may be defined as independence. Of course multitudes of women have had to work in various ways in humble capacities, and have so managed to keep the wolf from the door, or at least to contribute to the upkeep of the family to which they have belonged. We are not dealing with this class of woman worker, nor shall we consider to-day the case of the mechanical worker who of late years has rendered a service to mankind by undertaking many clerical duties.

The woman worker we have in view is the educated woman who has been brought up in comfort, if not in luxury, and who has every qualification to make her a social success. Women of this type, when means fail, are to be pitied, because tradition and convention both compel them to consider that in their case work, if not discreditable, is at least undesirable, and a thing to avoid at all costs. Why the English nation should cling with such tenacity to this wrong and ridiculous view of a woman's mission, in the circumstances named, it is difficult to say. The result of this tradition has often proved a calamity and led to tragedies in families, resulting in misery to individuals which ought never to have been possible. The inculcation of such false sentiments in regard to work for educated women in the past has unfitted them by inheritance to attempt the difficult task of facing the world and maintaining themselves by their own work with honour and in comfort. What this inherited tradition has caused the English nation to lose in the past it would be difficult to over-estimate. Its immediate effect in the present is that every educated gentlewoman, who finds herself suddenly called upon to strive by work to maintain herself in independence, is greatly handicapped for the task. The majority of such women are educated in a haphazard sort of way, and are not prepared to face life in a practical manner as they ought to be. In upper and middle-class families everything is sacrificed to secure the education and training of the sons, whilst the girls suffer neglect in both these respects.

When the educated woman of the class we are considering is called upon to face the world, and to work in order that she may live and acquire a position for herself, she finds herself lacking in the very elements which are essential to success. If she should be fortunate enough to have at her disposal the counsel and support of those who are able to tide her over the initial difficulties, and to put her in the path where such talents as she may possess are best calculated to enable her to reach the desired goal, she may, by resolution and dogged perseverance, attain it ultimately.

One essential to success is that the woman worker shall be self-contained and free to devote her whole energies and strength to the accomplishment of the object which necessity or individual talent has set before her. Unless the woman worker can be freed from the social ties and entanglements which attach themselves to the majority of upper and middle-class women, who not being workers would otherwise find it difficult to exist at all, her efforts, like the corn sown among thorns, are rendered futile, and must in fact fail, however great may be her energy and determination. Such impediments are due of course to the fact that the mother has inherited all the prejudices of the social sphere in which she was born, and that she is so incapable in most cases of realising the importance of shielding her child, who has to work for her living, from the burrs and obstacles which we have just referred to. Hence the initial start may, and in fact does prove fatal to a large number of educated women, who might otherwise succeed as women workers.

Again, when such difficulties have been successfully surmounted, and the educated woman worker is fairly on her way to secure an income and a position, she is often stopped or hindered by a recrudescence of the difficulties raised by her family or her parents, who consider that they are justified in appealing to their child's affection for themselves, in order to minister to their own comfort by trying to bring her once again under their immediate control in a household, where the earnings of the woman worker would be expended largely in promoting the comfort of the family at the expense of the worker. Experience proves how lamentable such inconsiderate selfishness may prove in its results. It cannot be too strongly enforced upon all women workers, that their first duty, where necessity has compelled them to face the world, is to provide that they shall make themselves secure and safe so far as possible. Until they have attained a position of strength and security it is nothing short of suicidal for them to permit any family ties of any kind to interfere with their progress, or to imperil their future. Resolution in this respect means security for all.

A woman artist, an authoress, or a woman who becomes a member of a learned profession, to succeed must, like the man, learn to stand in her own shoes. She must have her little home or chambers, and everything connected with them must be so arranged as to secure that her business shall be furthered and not hindered, and that the work shall be the first consideration in everything. For women workers, as for all, individual independence is the first essential step on the road to individual success. No appeals based upon family ties or necessities can safely be accepted by an educated woman worker, as sound reason why she should handicap herself, so far as her work is concerned, by sinking her independence in the household arrangements of her family when she is beginning to earn an income. Any such claims should be satisfied by a contribution in cash to a definite amount well within her means, which can be applied by the family to their upkeep at their discretion, and so her independence will be maintained and all such appeals may be properly met. It is a lamentable thing to realise that the woman worker should be called upon, by appeals to her feelings, to have to exhibit qualities which no man has to face, and to provide an added store of energy beyond the needs of her work, in order to keep the progress of her professional life free and independent. We hope, as the world grows older, and society becomes accustomed to the advantages and claims of the educated woman worker, that she may one day come to be recognised, as having at least equal claims with the sons, to an adequate education, and to the co-operation of the family to help and not to hinder her from the outset of her career in promoting its success to the utmost possible extent.

The answer no doubt which the world will give to these contentions is that women are not at present ripe for changes of this kind. The majority of women cannot and will not give up the old family ties when called upon to face the world as educated workers. Hence the attempt of the majority of women in this direction may continue to fail for another generation. The reply to this is simple and complete. Every educated woman worker who follows the course we have indicated, and attains a position of independence and security for herself, will then be able to give strength to her family, whilst she fulfils to an extent, infinitely more

THE WOMAN WORKER—*Continued.*

valuable and complete than was ever possible before, every legitimate tie which the family can properly demand at her hands. In fact, the woman worker so circumstanced becomes a tower of strength to her family, whilst she is a source of infinite comfort to herself. All that society and educated women who may have to work have to realise is that a daughter, like a son, who has to face the world and to win her way in it, must from the outset of her career be absolutely independent and free from every tie which may tend to hamper her in her work. Once this security is attained, then strength will flow from the woman worker to all who are nearest and dearest to her, and so this period of relative isolation and absolute independence, whilst it is essential to success, may, and indeed in our view must, tend to raise the character of all such family ties, and to make them infinitely more precious in fact than they have ever been before.

The plain truth is that the selfishness of parents, possibly of husbands, sometimes, and of the least successful members of families, based on the traditions of the past, when educated women workers in this country were unknown, if allowed to go unresisted and unchecked, must kill the success of any working member, being a woman. Unless she has character and outside support enough to enable her to see the humbug of the spurious claims set up by so-called family ties, they must, if admitted, destroy the educated woman worker and may ultimately bring the family to destruction and misery too. Fathers and mothers, when their fortunes are at a low ebb, are too apt in this country to adopt methods, and endeavour to exercise influences over the few members of their families, being women, who make a supreme effort to attain an independent position as workers, which, if applied by ordinary people to ordinary workers, would be regarded, as they indeed are, as nefarious and abominable. How many hundreds of women within our knowledge, who at great personal cost have learnt their business and obtained a position and an income upon which they can live in comfort, have been so preyed upon by their families that their savings have been exhausted, and when at last their strength has given out they have found themselves without means, reduced to a position of dependence upon the charity of others, or even left to the mercy of the Poor Law! Down this road no educated woman worker who makes up her mind to face the world, and to attain independence, should ever allow herself to be forced by wrongly yielding to the demands of so-called family ties. Such specious claims could never be raised if her own people had a real self-denying, honest affection for the educated woman worker and genuine admiration for her devoted efforts. Hence, whenever these claims are made they should be regarded by her as a temptation to which she must in justice ever turn the deafest of deaf ears.

### Presentations.

Miss A. N. Ferguson on leaving the Northern Counties Hospital for Incurables, Walmersley House, near Bury, Lancashire, after a short period of duty, received from the patients a handsome silver serviette ring, with her monogram, and the following inscription: "To Sister A. N. Ferguson, as a token of affectionate esteem, from the patients, Walmersley House, September 1st, 1905." From the staff Miss Ferguson received a handsome silver rose bowl, bearing the following inscription: "To Sister A. N. Ferguson, as a token of esteem from the staff, Walmersley House, 1905."

### "The Hospital" Convalescent Fund.

The Hon. Sec. acknowledges, with thanks, the receipt of 2s. 6d. from the Travel Correspondent.

### Incidents in a Nurse's Life.

**Contributions for this column are invited.**

### NURSING AMONG MILL OPERATIVES.

Some little time ago I was sent from the nursing home with which I was connected to nurse a case of scarlet fever. The address was in a crowded back street in one of the busiest quarters of a northern town. Arriving I found my three patients, a mother and two children, all very ill; the mother suffering from heart disease, as well as scarlet fever, and the children delirious and restless. All had been in bed for a week. During that time none of them had been washed, the room was filthy, and the window, of course, carefully closed. Before the doctor arrived I got the room into something like cleanliness, washed the patients, who thought they must surely die, and opened the window to let in a little fresh air. When the doctor appeared, he said: "Will you stay, nurse? It is a pigsty, and I don't know what you can do; but nearly every house has a case or more, and we cannot isolate properly, for the hospital is full." I replied that I had come to stay, and would do my best. It was hard work, there was nowhere to sleep—no one had thought of that, although the people being mill hands and earning good money were going to pay for a nurse themselves. The father, two sons, and one daughter went daily to the mill, notwithstanding the risk of infection.

#### Restricted Sleeping Quarters.

For the first two nights I could not leave my patients, but the third night I made up a bed in a tiny room not much bigger than cupboard, and there I slept for the whole seven weeks. When all had gone to their work each day, I went downstairs and cooked and prepared my own and my patients' meals, and often washed up and cleaned down the home, for the daughter was a most dirty, thriftless creature. One Sunday I went out for my walk and when I returned I found nine people in my patients' room, the window nailed down, and the husband declaring that I should not open it again. However, I got my way, cleared the room, and let in more fresh air. In time they all enjoyed it and washing was looked forward to with delight.

#### How I Made the Plum-pudding.

It was winter, and on Christmas Day my two small patients were able to get up. The two sons had said they should get no pudding this year, so two days before Christmas I went out and got ingredients and made a good plum-pudding and gave it its first boiling. Then, on Christmas morning as all were at home, of course I could not go downstairs amongst them, I told the girl to put the pudding into boiling water and boil for two more hours, feeling quite happy that the boys would have their pudding. But, alas, to my disgust about 12 o'clock I smelt burning and ran down to find the girl had gone out, leaving the dinner to take care of itself, so that the pudding was completely spoilt and the bottom nearly burnt out of the saucepan which had contained it. This is typical of the mill-girl; they earn good wages but do not know how to get comfort with their money, the waste and the thriftlessness is terrible. At the end of seven weeks I returned to the home, having sent all my patients to convalesce in the country, not sorry for my experience in the slums but still glad to get back to comfortable sleep not disturbed by street fights and brawls every night, and how I enjoyed the invitingly cooked food! I have nursed wealthy patients and middle-class and have always got on well, but I think I enjoyed nursing my little slum children as much as any other cases I have had. The mother was most grateful, and after a time the father seemed less inclined to get drunk than he was at first. So I felt that on the whole my time had not been wasted.

### A Book and its Story.

### AN ANGLO-INDIAN ROMANCE.

In "Rose of the World" Mr. and Mrs. Egerton Castle take their readers to India, and it is there that the fascinating heroine Rosamund Geradine, wife of the Lieutenant-Governor, Sir Arthur Geradine, is living when the book opens. She had married him some years previously, when a young widow. He was approaching the prime of life at the time. "The great man had chosen her in the zenith of his life and success because of her beauty. She had little birth to boast of, and no fortune. But it pleased him at every turn to trace in her those points which are popularly supposed to belong only to the patrician." Sir Arthur, encased in oblivious self-satisfaction failed to realise the possibilities for joy and suffering that lay dormant in the widow of Captain English. Under his courtly grace of manner there lay an immovable desire to bend all things to his ruling, and we read: "No one would have been more surprised than Sir Arthur himself had he been told that he was a tyrant. It was only after experience that people felt the steel behind the velvet glove. . . . Such a character combined with a mighty intellect would have been an enormous power for good. Unfortunately, it was upon the slightest premises and with limited reasoning faculties that Sir Arthur formed his unalterable views of life." His niece, Aspasia Cuningham, is a frankly delightlul study of a girl. Devoted to music, with aspirations towards a professional career, she had, on the death of her mother, to give up these dreams cf future distinction and to come out to India to her guardian and uncle. Throughout the pages of this dramatic book Aspasia flits in and out with youthful irresponsibility, adoring her aunt, and keeping a critical and irreverent eye upon "The Runkle," a name that she had given to Sir Arthur in return for "Raspasia" the one by which he, to her annoyance, addressed her. He will always say, "My dear Raspasia . . . My dear Raspasia! So I have got into the way of calling him: My dear Runkle Rarthur!" and now—it's really quite wicked—everybody calls him the Runkle, all the secretaries and things—behind his back, of course. Her frank young laugh rang out infectiously." Upon some occasion when Sir Arthur, "benevolent, consequential," had been officiously attentive to Lady Geradine who was only asking to be left alone, Aspasia, a thousand shades of exasperation and scorn upon her expressive countenance, had exclaimed: "If ever there was a woman killed by kindness, it is you, poor Aunt Rosamund!" and flinging her arms round the still figure: "Oh, darling," she whispered, with the wail of an ever renewed complaint, "Why do you always *always* give in!"

Lady Rosamund gently disengaged herself, bringing her eyes back from the distant loveliness with a perceptible effort. "Oh! Baby," she said in a tone of melancholy mockery, "when you have lived as long as I have you will see how much simpler it is."

Lady Geradine's first husband, Captain Harry English, had been a distinguished soldier, and the task of writing his life had been entrusted to an old friend and fellow comrade Major Raymond Bethune, who had fought with him in the last days at Inziri. "Raymond Bethune, major of Guides, loved the fierce lads to whom he was at once father and despot, as perhaps he could have loved no troop of honest thick-skulled English soldiers. . . . His duty sufficed him. He found happiness in it, that it was his duty. Such men as he are the very stones of our Empire's foundation." Major Bethune in pursuit of further material for the life of Captain English had called upon Lady Geradine. While awaiting her entrance he makes mental notes of his surroundings. . . . "He put down his hat and looked round. Not a hint of tropical colour, not a touch of exotic fancy, of luxuriant art anywhere; but the green and white of rosebud chintz, the spindle-legged elegance of Chippendale, the soft note of Chelsea china, the cool grey and whites of Wedgwood. . . . Such a room, dignified with the consciousness of a rigid selection, reticent to primness in its simple yet distinguished art, fragrant with the *pot pourri* of English gardens, fragrant, too, with memories of generations of wholesome English gentlefolks you may meet any day in some old manor house of the shires. To transport the complete illusion to the heart of India Bethune knew well must have cost more labour and money than if the neighbouring palaces had been ransacked for their treasures. . . . A soft murmur of muslin skirts against matting grew into silence. Lady Geradine came into the room. . . . So this was she—Rose of the World! Not so beautiful as he had fancied. And yet, yes—grudgingly he had to admit it—beautiful, and more. With every instant that passed, the extraordinary quality of her personality made itself felt upon him; and his heart hardened. This grace more beautiful than beauty; those deep strange eyes startling with their unexpected colour, green-hazel; in the pallor of the face under a crown of hair fiery gold. . . . Oh, aye, that was she! Even so had Harry English described her to his only friend. . . ." "Major Bethune?" she said, questioningly, as she approached. "My name must be familiar to you," he replied gravely. She paused a second, slightly contracting her brows; then shook her head, with a smile. "I am afraid—I have such a bad memory. But I am very glad to see you." She put out her hand graciously. He barely touched it with his fingers. "Pray sit down," she said; and took her own chair. One felt the accomplished woman of the world. No awkwardness could exist where Lady Geradine had direction of affairs. Sweet, cool, aloof, the most exquisite courtesy marked her every gesture. Had the newcomer been shy he must promptly have felt reassured; for a long looked for quest could not have been more easily welcomed. "Have you been long here?" She seemed to take his visit as a matter of course. "I arrived yesterday; I am on leave." "Indeed, and what regiment?" He told her. A change, scarcely perceptible, passed over her face. She compressed her lips and drew in her breath, a trifle longer than normal, through dilated nostrils. Major Bethune was smarting under Lady Geradine's apparent forgetfulness, he reads indifference and disloyalty to the memory of his dead comrade in her bearing. "I am afraid," he said briefly, "that my presence must seem an intrusion. But I trust you will forgive me when you understand upon what errand I come." . . . "The fact is," he went on doggedly, "I have been asked to write a life of your husband." "But the Runkle is not dead yet, worse luck!" The last exclamation was fortunately not an audible one. Lady Geradine was smiling again in her detached manner. "A great many people, distinguished people, Baby, have their lives written before they die, and they then have the chance of correcting the proof sheets. I daresay your uncle will not object." Major Bethune allowed a pause to fall before continuing his speech; then he said, with almost cruel distinctness: "I beg your pardon, Lady Geradine, I should have said your late husband; I refer to Harry English." The development of the drama in which Rosamund Geradine takes the leading part starts frcm this point. Aspasia Cuningham supplies the conscious and Sir Arthur and the Lady Aspasia the unconscious humour to a book which is written with distinction. On the whole the dominant note is a tragic one, but the curtain goes down on the reunion of lovers, and the end is peace.

* "Rose of the World." By Agnes and Egerton Castle. (Smith Elder and Co. 6s.)

### The Railway Accident at Witham.

### HOW THE NURSES WORKED.

The advantage of having small nursing institutions scattered throughout the country was once again demonstrated on Friday last when the terrible railway accident at Witham taxed the resources of the neighbourhood to the uttermost.

The St. Albans Diocesan Nursing Institution has its headquarters at Witham, and the matron taking holiday duty there had jokingly remarked only the day before the accident occurred that she hoped something would happen to employ her nurses for they were coming in fast.

The something did happen, and for forty-eight hours every available pair of hands, including the matron's was pressed into the service, whilst three of the nurses who were away on holiday were summoned by telegraph.

Help was also rendered by the district nurse from Hatfield Peverel, who bicycled over immediately on hearing the news, by one of the Witham Cottage nurses, and by a nurse from the Sussex County Hospital at home on holiday.

A slight mitigation of this awful tragedy was the fact of the train being derailed actually in the station, so that help was instantly forthcoming and no time was lost in rescuing the sufferers. Another was the admirable self-possession of all concerned. A helper, arriving on the scene half-an-hour after the accident had occurred, could at first hardly realise the magnitude of the catastrophe, so complete was the absence of fuss or excitement. There was no crying, no shrieking, and no helpless wringing of hands.

The uninjured passengers began at once to assist the wounded; water and sponges, lint, splints and bandages, appeared as if by magic, an army of willing bicyclists were in readiness to fetch anything that was asked for, and the doctors with their band of trained and untrained assistants set to work as coolly as though a railway accident were an everyday occurrence. The only thing that hampered them was the over-eagerness of the residents to open their doors to the sufferers.

The wounded, as they were released, were to have been conveyed on litters to a large building—the Corn Exchange—near the station, whence they could easily be passed into the Colchester Hospital, but several were carried into neighbouring houses and up such narrow staircases that it was impossible to bring them down again. The consequence was that for a time no one knew what had become of them, and it was a long while before the doctors could gather for how many patients they were really responsible. It also complicated the subsequent nursing and most of the nurses were at work for 24 hours at a stretch, so many patients requiring attention all night.

Five of the most serious cases are still being nursed by the Diocesan Institution, the rest were either sent to Colchester or Chelmsford Hospital, or returned home after a day or two's rest.

The next curious fact about the wounded was the almost entire absence of crushing or mangling and the immunity of the lower limbs. Nearly all the injuries were to the head or upper limbs, and the number of fractures was comparatively small. The marvel is that so few of the passengers were really damaged.

An inspection of the wreckage immediately after the accident made it seem almost impossible that any one in or near the train could have escaped alive. It was an exciting and exhausting 24 hours for all concerned, and Withamites may well be proud of the coolness, ability, and kindness displayed on all sides. Doctors, nurses, police, railway men and residents vied with each other in their untiring devotion. Meals were forgotten and the partridges had another day's grace; everyone was possessed with the desire to help, and English pluck and English humanity were amply vindicated that day.

### Appointments.

**No charge is made for announcements under this head, and we are always glad to receive and publish appointments. The information, to insure accuracy, should be sent from the nurses themselves, and we cannot undertake to correct official announcements which may happen to be inaccurate. It is essential that in all cases the school of training should be given.]**

Bideford Hospital and Dispensary.—Miss Lizzie White has been appointed matron. She was trained at King's College Hospital, London, and has since been nurse at the East London Hospital for Women, Shadwell.

Chorlton Union Infirmary.—Miss Sophia Maud McKinna has been appointed charge nurse. She was trained at Poplar and Stepney Sick Asylum.

Derbyshire Royal Infirmary.—Miss Jean J. Anderson has been appointed sister. She was trained at Coventry and Warwickshire County Hospital, where she has since been sister and night sister.

East and West Suffolk.—Miss Caroline Swain has been appointed inspector of midwives. She was trained at the Royal Hants County Hospital, Winchester, and at Queen Charlotte's Hospital, London. She is registered under the Central Midwives Board.

Grimsby District Hospital.—Miss Fanny Barnes has been appointed sister. She was trained at the Royal Free Hospital, London, where she has since been acting night sister, and charge nurse of the casualty and out-patients' departments.

Jessop Hospital for Women, Sheffield.—Miss Mary E. Davison has been appointed staff nurse. She was trained at the City of London Lying-in Hospital.

Patricroft Infirmary, Barton-in-Irwell Union.—Miss M. J. Keary has been appointed charge nurse. She was trained at Ecclesall Bierlaw Union Infirmary, where she has since been temporary charge nurse.

Plaistow Fever Hospital.—Miss Elizabeth Mary Radcliffe has been appointed Sister. She was trained at Sunderland Infirmary, and the Sanatorium, Cardiff. She has since been Sister at Darlington Fever Hospital, and has done private nursing.

Rochester Workhouse Infirmary.—Miss Gertrude Evans has been appointed superintendent nurse. She was trained at St. George's Infirmary, Fulham Road, where she was afterwards sister.. She has also been night superintendent and sister at Fulham Infirmary, Hammersmith, and sister at Croydon Infirmary.

Royal Surrey County Hospital, Guildford.—Miss E. M. Plomby has been appointed sister of the children's ward. She was trained at Sussex County Hospital, Brighton.

St. Monica's Hospital for Children, Brondesbury Park, N.W.—Miss May Christmas has been appointed matron. She was trained at West Ham Hospital, London, E., and has since been staff nurse and sister at the Jenny Lind Hospital, Norwich, night charge nurse at the Children's Hospital, Paddington Green, W., and sister at St. Monica's Hospital.

Starcross Nursing Association.—Miss Lilian M. Francis has been appointed district midwife and cottage nurse. She was trained at the Stapleton Infirmary, Bristol.

Totnes Cottage Hospital.—Miss Lucy M. Gowan has been appointed matron. She was trained at the Buchanan Hospital, St. Leonards, and has been nurse at the Camberwell Union Infirmary.

### Everybody's Opinion.

[Correspondence on all subjects is invited, but we cannot in any way be responsible for the opinions expressed by our correspondents. No communication can be entertained if the name and address of the correspondent are not given as a guarantee of good faith, but not necessarily for publication. All correspondents should write on one side of the paper only.]

### LADY DOCTORS AND MIDWIVES.

"J. W." writes: Another "C. M. B." no doubt holds a post with a stated salary and knows nothing of getting a living by district midwifery. The competition gets keener every year. Referring to lady doctors and medical students from hospital, I should like to say that they are all looked upon by the poor as fully qualified, and if they can be attended to by the lady doctors for the fee of 5s. it most certainly does harm to midwives, as they have to charge a nominal fee to compete with them.

### AN ISOLATION HOSPITAL WITHOUT A TRAINED NURSE.

"M. C. T." writes: Having read about "Village Nursing in Northampton" I do not know if the Local Government Board are aware that an isolation hospital in this county, which contains 80 beds, is not only without a trained matron but also without a trained nurse. Before Northampton thinks of getting trained nurses for agricultural localities, I think that a trained nurse should be got for this hospital. I was there nearly two years; since I left they have done without a trained nurse paying untrained women at the rate of £26 to £40 per annum. There is no resident doctor. On several occasions I had to get up at night to different patients, one a tracheotomy case, and the doctor said if it had not been for the constant attention I was able to give the child could not have lived. The assistant nurses did not know how to clean the tube.

### TRAINED NURSES ON SEA-GOING VESSELS.

"Sister" writes: I have been interested in reading the article "Trained Nurses on Sea-going Vessels." I joined the Royal Mail Steam Packet Company last November, and was sent out on station to the West Indies for six months during the tourist season. I nursed about six cases, *i.e.* hæmoptysis dysentery, appendicitis, tonsillitis, several cases of "mal de mer," and a passenger who required fomenting every four hours. I and another nurse, who had been stationed on the *Eden*, returned home on the *La Plata*; we were called upon to nurse the operation case mentioned in The Hospital. The other nurse did night duty and I day. Both operation and nursing were done under great difficulty owing to the rough voyage and want of appliances. If it had not been for the skilful surgeon on board and proper nursing the patient would never have reached Plymouth. He was a soldier who had been out to Jamaica for three years. But for us it would have been impossible for him to have been nursed, as the ship's nurse had to do the work of stewardess. Unless shipping companies carry a nurse independent of the stewardess I am afraid it will never answer. A trained nurse will not undertake the duties of a stewardess. While out on station I only arranged the flowers for tables and attended on those who were sick, therefore I had a very good time.

### NURSES ON HOLIDAY.

"A Nurse" writes: It may interest some of your readers to know of a very pleasant watering-place in the highlands of Scotland. "Strathpeffer," or "The Strath" of the Peffery, is a green valley on the verge of a wild and rocky hill country, the scenery of which is very charming and consists of hills covered with trees and heather, small fields, and white-washed cottages dotted here and there. Many people visit the place during the summer months to take the sulphur and chalybeate waters, peat and other baths. The waters are taken from 6.30 to 9 a.m. and 12 to 2 p.m., and 4.30 to 5.30 p.m. Baths are open to ladies from 8 a.m. to 7 p.m., and to gentlemen from 7 a.m. to 7 p.m. The hotels are very expensive, as also are most things. The small houses take boarders at a cheaper rate, but they are comparatively dear. Amusements at Strathpeffer consist of drives to the various places of interest, a band near the pump-room playing several hours of the day, concerts and games in the pavilion. Very few people are in the Strath during winter as there is no attraction then. I was fortunate enough to go to this charming place in August last, and travelling by night, I left Euston at 7.45 p.m., arriving at Inverness at 9.15 a.m., where we waited half an-hour and then left for Strathpeffer, arriving at 11 a.m. My visit was most beneficial to myself, and I returned to my duties feeling refreshed in body and mind.

### THE REGISTRATION OF NURSES.

"A Scotch Certified Male Nurse" writes: I am a constant reader of The Hospital, and I am in the hope that all nurses who take an interest in the welfare of their calling will speak out when they have the opportunity of doing so concerning the employment of certified and unqualified male and female nurses, as to which I know a good deal can be said. A few male asylum men calling themselves The ——— Male Nurses' Association, have persuaded a gentleman here to act as a kind of registrar for them and provide them with cases when notified. A good many of these men have never served in either a hospital or an asylum, and, to my knowledge, have had no training whatever. May I ask you if it be right that doctors should be imposed on by such shams? I will give you an instance: one of the male nurses referred to, was a doctor's coachman, another a butler's footman, a third a hospital gateman, and yet a fourth a hospital kitchen porter. Can it be expected that these men will give an intelligent report of any kind to the doctor in charge of the case? From my experience a nurse's duty, if properly discharged, is of a very responsible kind. What do these men know of the management of the bodily condition; management of the mental condition; condition of the will; changes in the feelings and instincts; suicidal cases; exaltation or perversion of mind; diseases and disorders of the nervous system; dementia, epileptics, arteries, bandaging, etc.? I admit that there is great improvement in the nurses nowadays, and that the report of the Select Committee on Registration of Nurses is in the right direction. A nurse ought to be certified and registered. I have no wish to say any more on the matter just now, as I think I have said enough to see plenty of replies about the registration of nurses soon in The Hospital.

### TRAVEL NOTES AND QUERIES.

By our Travel Correspondent.

Accommodation in Southport (Stons).—Many thanks for information and address. Both have been filed for future use. Unfortunately, I fear the terms are beyond the purses of most of our correspondents, as I presume the sum stated did not include food. If I am wrong, kindly correct me.

The Canary Islands (Ishtal).—These are the hotels you ask for (all, I fear, somewhat expensive):—The Hôtel Métropole, Las Palmas. Terms, £3 10s. per week, but a few rooms at £2 16s. This is a charming house. The Hôtel Aguere and Continental, at Laguna. Terms, 8s. to 10s. per day. Hôtel Martianez, at Port Orotava. The same terms. As you have time before you, write at once to the various proprietors and ask their lowest terms at the date required. The cheapest usual route is by the Elder, Dempster, and Company's steamers from Liverpool. First class £10, or first-class return (available for 12 months) £15. A cheaper way is by the African Steamship Company from Liverpool. Write and ask the fares.

Rules in Regard to Correspondence for this Section.—All questioners must use a pseudonym for publication, but the communication must also bear the writer's own name and address as well, which will be regarded as confidential. All such communications to be addressed "Travel Correspondent, 28 Southampton Street, Strand." No charge will be made for inserting and answering questions in the inquiry column, and all will be answered in rotation as space permits. If an answer by letter is required, a stamped and addressed envelope must be enclosed, together with 2s. 6d., which fee will be devoted to the objects of "The Hospital" Convalescent Fund. Ten days must be allowed before an answer can be published.

### Notes and Queries.

**REGULATIONS.**

**The Editor is always willing to answer in this column, without any fee, all reasonable questions, as soon as possible.**

**But the following rules must be carefully observed.**

**1. Every communication must be accompanied by the name and address of the writer.**

**2. The question must always bear upon nursing, directly or indirectly.**

**If an answer is required by letter a fee of half-a-crown must be enclosed with the note containing the inquiry.**

*Nice.*

(184) Can you tell me where I can obtain particulars about the nursing for the Queen Victoria Memorial Hospital now being built at Nice?—*Nurse H.*

You might write to the British Consul, Nice. If this does not succed write to us again.

*Colonial Nursing Association.*

(185) Wishing to apply for a post under the Colonial Nursing Association, I shall be obliged if you will tell me how and where to apply for such?—*Sister.*

Will you kindly give me the address of the Colonial Nursing Association?—*M. M. J.*

Write to the Secretary, Colonial Nursing Association, Imperial Institute, London, S.W.

*Salary.*

(186) Will you kindly advise me what to do in the following case? I am a masseuse and have been working at an institution for four weeks. Last Monday I received a wire saying my brother was very ill, and I had to go home at once. I wired to the nurse in charge and told her that I had to go, and I also sent her another wire saying I should be back on Wednesday morning. I got back on Wednesday and found a letter saying she would not require my services any longer, and enclosed £1 1s., one week's salary. She owed me three weeks, as I had only been paid once since I had been there. I wrote asking her for the rest of the money and she replied saying I was not entitled to my salary. Can I claim for the two weeks I was working for her and also a week's salary in lieu of notice?—*A. W.*

You should have seen the nurse in charge and obtained her permission to go to see your brother. With regard to the amount of salary you are entitled to receive it would seem that you have a right to the fees for the other two weeks. But in any case you should interview the nurse in charge. It is a mistake for nurses to go to law if they can possibly avoid doing so.

*Monthly Nursing.*

(187) I venture to write and ask for your advice with regard to getting into a nursing home to work from as a monthly nurse. I am certificated under the Central Midwives Board, but have had no other training. I could only afford a short time for training as it is very necessary I should earn as much as possible, but I find my lack of general training prevents me entering a nursing home. I have answered so many advertisements and it is always the same story. Can you let me know of any institution I can join under these circumstances.—*Gyles.*

We can only suggest that you should advertise, reply to advertisements, or write to any institution which you think might possibly suit.

*Register of Cases.—Midwives Act*, 1902.

(188) In the book of register of cases which I keep it says:—"Duration of first, second, third stage of labour." I should be glad if you would advise me what to fill this up with, as I have a lot of cases and several I have attended lately have been delivered within an hour after my arrival. What should you put for the first, second and third stage in a case like that? I should be glad of the benefit of your advice as to how I should fill this up generally.—*Myra.*

The first stage of labour is from the commencement of definite pains to full dilatation of the cervical canal and os uteri. The second stage lasts from the end of the first stage to delivery of the child. The third stage is from the end of the second to delivery of the membranes and placenta. You should time these different stages and enter the results in your register. If you are not present at any one of them you should say so in your register and mention the patient's estimate of the first and second taken together. You will nearly always be able to observe the third stage yourself.

**Handbooks for Nurses.**

| | Post Free. |
|---|---|
| "How to Become a Nurse: How and Where to Train." | 2s. 4d. |
| "Nursing: its Theory and Practice." (Lewis.) ... ... | 3s. 6d. |
| "Nurses' Pronouncing Dictionary of Medical Terms." ... | 2s. 0d. |
| "Complete Handbook of Midwifery." (Watson.) ... | 6s. 4d. |
| "Preparation for Operation in Private Houses." ... | 0s. 6d. |

Of all booksellers or of the Scientific Press, Limited, 28 & 29 Southampton Street, Strand, London, W.C.

### For Reading to the Sick.

A GARLAND OF PRAISE.

A wreathed garland of deserved praise,
Of praise deserved, unto Thee I give,
I give to Thee, who knowest all my ways,
My crooked winding ways, wherein I live,
Wherein I die, not live; for life is straight,
Straight as a line, and ever tends to Thee,
To Thee, who art more far above deceit,
Than deceit seems above simplicity.
Give me simplicity; that I may live;
So live and like, that I may know Thy ways,
Know them and practise them: then shall I give
For this poor wreath, give Thee a crown of Praise.

*George Herbert.*

This joy in obeying, this happiness in the sense of Christ's help, this cheerfulness in the sight of God and man, is one of the great missionary powers on earth, second only to the power of love. And if we would ask how, without any ostentation, we can best obey our Lord's command to "let our light so shine before men that they shall glorify our Father in heaven"; how we can combine such a command with the direction "not to let our left hand know what our right hand doeth"; the answer is, let all men read in your face the happiness of a Christian that loves his Master. Let them see in your unvarying cheerfulness the assurance of your faith, and the certainty of your hope, and the blessedness of your love.—*Bishop Temple.*

Men are for ever seeking after demonstrations of the truth of Christianity, and there shall no demonstration be given. Many stand outside the pale and ask for a reasoned philosophy and irrefragible proofs that Christianity is true, and say that when this has been vouchsafed they will enter the sacred precinct, and take the yoke upon them, but not till then. Such persons ask for that which neither our Lord nor His apostles, nor any of the old prophets, ever promised—for that which, according to their teaching, was, in the nature of things, impossible. "If any man will do His will, he shall know of the doctrine"—this was their language. . . . One touch of sympathy with the mind of the Divine Teacher makes many things plain which before seemed hard sayings and unbelievable.—*Principal J. C. Shairp.*

Think a little less of your sorrows and more of your joys, for the joys will make you grateful, and gratitude is in itself one of the most beautiful pleasures of the soul. And being grateful, you can take this blessing ("The grace of our Lord Jesus Christ be with you all") to yourself, and make it yours, for part of the grace of the Lord Jesus is to have a grateful heart.—*Stopford Brooke.*

Think not of what is from thee kept;
Think rather what thou has received:
Thine eyes have smiled if they have wept,
Thy heart has danced, if it has grieved.
Rich comforts yet shall be thine own;
Yea, God Himself shall wipe thine eyes;
And still His love alike is shown
In what he gives, and what denies.

*H. S. Sutton.*

## Figures and Tables

**Fig. 10. f1:**
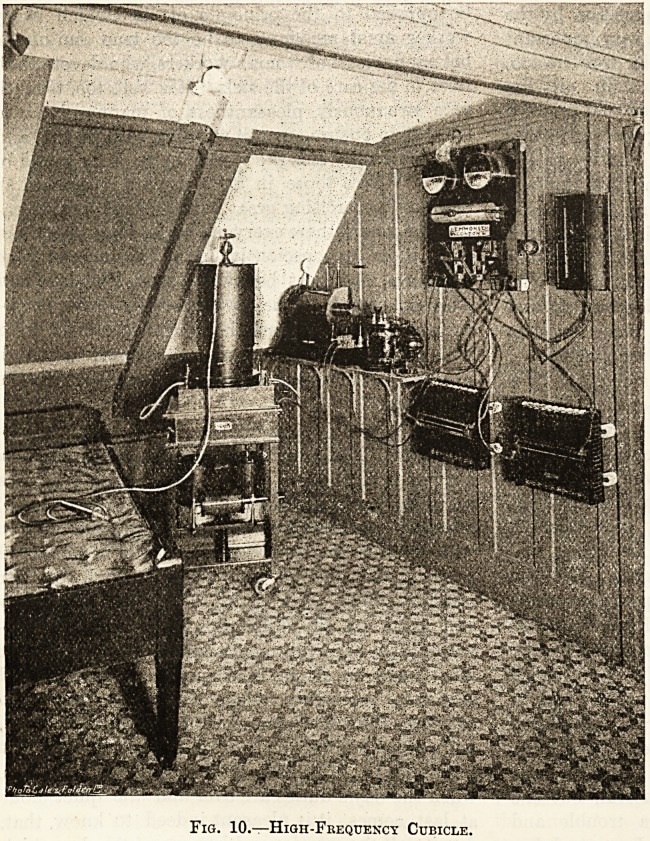


**Fig. 11. f2:**
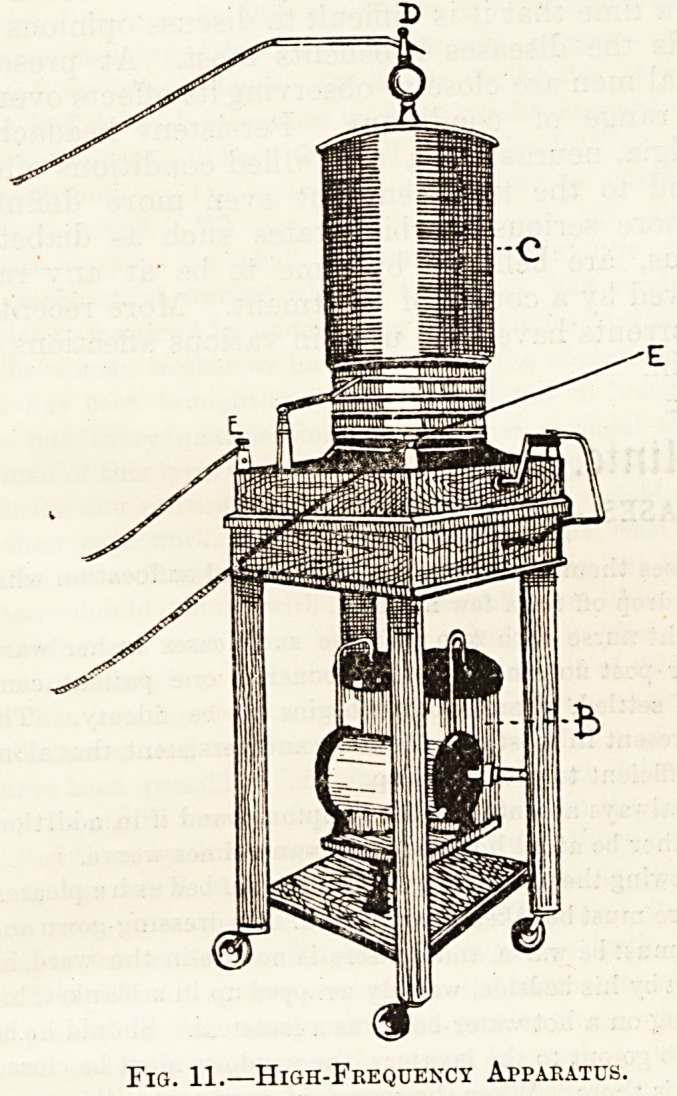


**Fig. 12. f3:**